# Antioxidant activity and protective effect of propolis against carbon tetrachloride-induced liver and kidney injury by modulation of oxidative parameters

**DOI:** 10.14202/vetworld.2021.3076-3083

**Published:** 2021-12-08

**Authors:** Redouan El-Haskoury, Noori Al-Waili, Zeineb Kamoun, Mohamed Makni, Ahmed Al-Waili, Badiaa Lyoussi

**Affiliations:** 1 Laboratory of Natural Substances, Pharmacology, Environment, Modeling, Health, and Quality of Life (SNAMOPEQ), Department of Biology, Faculty of Sciences Dhar Mehraz, Sidi Mohamed Ben Abdellah University, Fez 30000, Morocco; 2 New York Medical Care for Nephrology, Richmond Hill, New York, United States; 3 Laboratory of Toxicology-Microbiology, and Environmental Health (UR11ES70), Faculty of Sciences of Sfax, Higher Institute of Biotechnology of Sfax, University of Sfax, Tunisia

**Keywords:** antioxidant, carbon tetrachloride, kidney, liver, propolis, toxicity

## Abstract

**Background and Aim:**

Propolis has a protective effect against cellular damage caused by toxic agents such as drugs, metals, xenobiotics, and chemicals. The aim of this study was to investigate the antioxidant activity and the effect of ethanolic extract of propolis on carbon tetrachloride (CCl4)-induced oxidative stress on kidney and liver injury in rat.

**Materials and Methods:**

The study quantified phenol, flavone, and flavonol in propolis and assessed antioxidant activity using 2, 2-diphenyl-1-picrylhydrazyl, ferric reducing antioxidant power, and molybdate. The investigators used four groups of rats to study the effect of propolis on CCl_4_-induced toxicity. Propolis extract was given orally (500 mg/kg) for 12 days, and CCl_4 (_1 mL/kg) was administered intraperitoneally on day 5 of the experiment. Blood and tissue samples of the liver and kidney were collected on day 13 to measure biochemical and oxidative parameters. The parameters included malondialdehyde (MDA), protein carbonyl formation (PCO), advanced oxidation protein products (AOPP), catalase (CAT), glutathione peroxidase (GPx), reduced glutathione (GSH), and ascorbic acid (AA). Biochemical parameters included liver enzymes, blood urea (BU), creatinine, and uric acid (UA).

**Results:**

CCl_4_ decreased antioxidant agents, including CAT, GPx, GSH, and AA in the liver and kidney tissues. The oxidative agents’ levels, including MDA, PCO, and AOPP, increased by CCl_4_ compared to the control group. CCl_4_ increased liver enzymes, UA, BU, and creatinine in the blood samples. Propolis significantly alleviated liver and kidney function, improved antioxidant parameters, and decreased levels of oxidative agents.

**Conclusion:**

The data showed for the 1^st^ time that Moroccan propolis has a protective effect against CCl_4_-induced kidney and liver toxicity by maintaining the activity of the antioxidant defense system, which was most likely due to its antioxidant activity.

## Introduction

Carbon tetrachloride (CCl_4_) is one of the most chemical substances widely used in the laboratory to induce experimental kidney and liver injury. Its toxicity is due to the generation of reactive free radicals, particularly trichloromethyl radical and trichloromethyl peroxyl radicals, which interact with membrane lipids leading to organ damage [1,2]. Accordingly, the neutralization of free radicals generated by exogenous antioxidants such as Vitamin C, flavonoids, and polyphenols prevents liver and kidney damage caused by CCl_4_ exposure [3,4]. We have recently found that propolis has a potent protective effect against paracetamol-induced liver and kidney damage [5]. Propolis is a sticky substance collected by honeybees on the buds of trees. The chemical composition of propolis is complex and influenced by the botanical origin, the harvest, and geographical origin [6,7]. It contains various chemical compounds, including flavonoids, phenolic acids, steroids, proteins, amino acids, vitamins, minerals, and inorganic compounds [7,8]. It has been shown that propolis collected from different areas has antioxidant and antimicrobial activities, antitumor, anti-inflammatory, immunosuppressive, immunostimulant, wound healing, radioprotective, anti-diabetic, and anti-Alzheimer activities [5-7,9-14]. Propolis has a protective effect on kidney and liver injury [15,16]. Propolis extract decreases urinary protein excretion and alleviates liver and kidney function deterioration caused by ethylene glycol ingestion in rats [11]. Furthermore, another study revealed that propolis contains polyphenols, including flavonoids, and demonstrates higher antioxidant activities than honey [13]. Propolis has a protective effect against 2,3,7,8-tetrachlorodibenzo-p-dioxin-, acute septic shock, and CCl_4_-induced hepatic toxicity; the result was most likely due to anti-inflammatory and antioxidant properties [17-19].

The composition of propolis is mainly dependent on its geographical location; therefore, its biological characteristics are closely related to a collection site’s characteristic vegetation. Although propolis protects the kidney and liver exposed to various chemical substances, its mechanism of action is not well understood. The significance of the study is to explore whether propolis collected from Morocco has a protective activity on liver and kidney injury caused by CCl_4_ administration. The study aimed to evaluate the antioxidant activity of the ethanolic extract of Moroccan propolis. Furthermore, the study investigated the protective effect of the propolis extract on CCl_4_-induced liver and kidney injury in rats and the impact of CCl_4_ on the generation of oxidative stress.

## Materials and Methods

### Ethical approval

The Animal Facility and the Laboratory of Physiology-Pharmacology & Environmental Health, Faculty of Science, Sidi Mohamed Ben Abdallah University, Dhar Mahraz, Fez, Morocco provided ethical approval (USMBA-PPSE ACU2016-03). The study followed the international principles for laboratory animals’ use and care, as found in the guidelines [20].

### Study period and location

The study was conducted in February 2017 at the Laboratory of Toxicology-Microbiology and Environmental Health (UR11ES70), Faculty of Sciences of Sfax, Higher Institute of Biotechnology of Sfax, University of Sfax, Tunisia.

### Hydro-alcoholic extract

The investigators collected propolis samples from Sidi Slimane city (West of Morocco). Propolis extract was prepared according to the method described by Miguel *et al*. [21]. One gram of the propolis sample was chopped into small pieces and extracted with 30 mL of ethanol (70%), under agitation (200 rpm) for 96 h at room temperature (22°C±02), and then filtered and evaporated until a constant volume using a rotavapor.

### Bioactive compounds quantification

Total phenolic content was determined according to the method described by Singleton and Rossi, with slight modifications [22]. Briefly, the technique included mixing 100 mL of an ethanolic extract of propolis or standard with 500 mL of Folin–Ciocalteu reagent solution for 6 min, and then 400 mL of 20% sodium carbonate solution were added to the mixture. The absorbance was measured at 760 nm after 2 h of incubation at 22°C±02. The total phenolic content was expressed as mg of gallic acid equivalents per 100 g of extract (mg GAE/100 g).

The flavone and flavonol content was quantified according to the method described by Miguel *et al*. [21]. Briefly, the technique included mixing 500 mL of an ethanolic extract of propolis or standard with 500 mL 2% aluminum chloride-ethanol solution. The absorbance was measured at 420 nm after 1 h of incubation at 22±2°C. Quercetin was used as a standard, and the total content was expressed as mg quercetin equivalents per 100 g of extract (mg QE/100 g).

### Antioxidant activity evaluation

The radical scavenging activity of the propolis extract against 2, 2-diphenyl-1-picrylhydrazyl (DPPH) free radical was measured using the method mentioned by Clarke *et al*. [23]. The method included mixing a 100 mL of ethanolic extract of propolis with 825 mL of a 100 mM solution of DPPH radical prepared in ethanol (96%). The absorbance of the solution was measured at 540 nm after 15 min of incubation in the dark at room temperature. The blank solution contained water instead of propolis extract was used. Several concentrations of samples were made. The half-maximal inhibitory concentration (IC_50_) (concentration of sample able to scavenge 50% of DPPH free radical) was determined. The test was performed in triplicate.

The FRAP of the ethanolic extract of propolis was determined using the method described by Saxena *et al*. [24]. The technique included mixing a 100 mL of ethanolic extract of propolis with 250 mL of 0.2 M phosphate buffer (pH 6.6) and 250 mL of 1% potassium ferricyanide. The mixture was incubated at 50°C for 20 min. Then, 250 mL of 10% trichloroacetic acid was added, and the mixture was centrifuged at 4000× g for 10 min. The absorbance was measured at 700 nm against a blank solution containing water instead of propolis extract. The test was performed in triplicate.

The total antioxidant capacity of the ethanolic extract of propolis was evaluated by the phosphomolybdenum method as described by Prieto *et al*. [25]. Briefly, the technique included mixing 0.2 mL of propolis with 2 mL of reagent solution (0.6 M sulfuric acid, 28 mM sodium phosphate, and four mM ammonium molybdate solutions). The absorbance was recorded at 695 nm against a blank sample. The measurement was expressed as equivalents of ascorbic acid/gram of the extract. The test was performed in triplicate.

### Animals and experimental design

The study included a total of 24 male rats weighing between 140 and 165 g. The rats were housed in cages at 25±1°C on a 12-h dark/light cycle, with free access to a diet and water *ad libitum* at the animal’s house, faculty of science of Sfax, Tunisia. The rats were divided into four experimental groups, six rats each, and treated as follows.

- Group 1 (Control); received distilled water (10 mL/kg.b.wt)
- Group 2 (Propolis); treated with ethanolic extract of propolis at a dose of 500 mg/kg.b.wt/day
- Group 3 (CCl_4_); treated with CCl_4_ on the 5^th^ day by intraperitoneal injection at 1 mL/kg. b.wt
- Group 4 (Propolis+ CCl_4_); treated with ethanolic extract of propolis (500 mg/kg.b.wt/day) and then received CCl_4_ on day 5 at 1 mL/kg.b.wt.

The distal water and propolis were used for 12 days, and gavage was used to deliver the interventions. CCl_4_ was injected intraperitoneally on day 5 after starting propolis or distal water treatment. Other studies used similar doses of propolis and CCl_4_ [26-28].

### Blood and organ samples

On day 13, the fasted rats for 12 h were sacrificed under ether anesthesia, and blood samples were collected from each animal, centrifuged at 3000 rpm for 15 min at 4C. The serum was isolated and stored at 20°C until biochemical analysis. The kidney and liver organs were quickly removed, cleaned, and washed in ice-cold saline solution. Each organ was finely minced and homogenized in phosphate buffer (0.1 M; pH 7.4) and centrifuged at 8000 g for 20 min at 4C. The supernatant was used to assay the oxidant parameters.

### Blood and biochemical analyses

Alanine transaminase (ALT), aspartate transaminase (AST), alkaline phosphatase (ALP), creatinine, blood urea (BU), and uric acid (UA) levels were assessed by enzymatic methods using commercial reagent kits from Biomaghreb (Ariana Tunis, Tunisia).

### Oxidative stress analysis in kidney and liver

Malondialdehyde (MDA) in the kidney and liver tissues was determined according to the method of Draper and Hadley [29]. The procedure included the spectrophotometric measurement of the color that occurred during the thiobarbituric acid reaction with MDA. The absorbance of the thiobarbituric acid-MDA complex was determined at 532 nm, and the content of MDA (nmol/g wet tissue) was calculated using tetraethoxypropane (standard).

Protein carbonyl formation (PCO) level in the kidney and liver tissues was measured according to the method of Reznick and Packer [30]. The principle of the assay involved the reaction between protein carbonyl and dinitrophenylhydrazine to form a 2,4-dinitrophenyl hydrazone product, which displays a maximum absorbance at around 370 nm. The concentration of protein carbonyls (nmol/mg protein) was calculated, using the molar extinction coefficient of DNPH, e = 22,000 M^-1^ cm^-1^.

Advanced oxidation protein products (AOPP) level in the kidney and liver tissues was determined according to the method of Witko *et al*. [31]. AOPP concentration was expressed as micromoles per liter of chloramine-T equivalents.

### Measurement of antioxidants in the kidney and liver

Catalase (CAT) activity was estimated according to Aebi [32]. A decrease in absorbance due to hydrogen peroxide (H_2_O_2_) degradation was monitored spectrophotometrically at 240 nm for 1 min, and the activity was calculated in terms of nmol H_2_O_2_ consumed/min/mg of protein.

Glutathione peroxidase (GPx) activity was measured according to the method described by Flohé and Günzler [33]. The action was expressed as nmoles of reduced glutathione (GSH) oxidized/min/mg protein.

GSH was measured by a spectrophotometric method using 5,5-dithiobis-2-nitrobenzoic acid, according to Ellman [34]. The assay depended on developing a yellow color when 5,5-dithiobis-2-nitrobenzoic acid was added to the compound containing sulfhydryl groups. The absorbance was measured at 412 nm, and total GSH content was expressed as micromoles per gram of tissue.

The ascorbic acid in the liver and kidney homogenates was determined by the spectrophotometric method of Jacques-Silva *et al*. [35] using dinitrophenyl-hydrazine. The absorbance was measured using a spectrophotometer at 520 nm, and the results were expressed as mmol/g tissue. All experimental investigations followed the BCPT policy for experimental and clinical trials [36].

### Statistical analysis

Values were expressed as mean±standard deviation. Significant differences between treatment effects were determined using a t-test and one-way analysis of variance, followed by Tukey’s multiple comparison tests.

## Results

### Total phenol, flavones, and flavanols content and antioxidant activity of propolis

The total phenolic content of the ethanolic extract of propolis was 1991.6 mg GAE/100 g. Flavones and flavanols contents were 328.3 mg QE/100 g of the propolis extract. The total antioxidant activity of propolis extract was 243.5±4.7 mg AAE/g. For antiradical power assessment, the IC_50_ was 38.1±23 mg/mL with the FRAP, and the IC_50_ was 33.1±0.9 mg/mL with the DPPH.

### The effect of propolis in CCl_4_ induced hepato-renal toxicity

In the CCl_4_ treated group, CCl_4_ caused a significant elevation of ALT, AST, ALP, creatinine, BU, and UA compared to the control group (Table-1). There was no significant difference between the propolis treated group and the control group, except the considerable lowering of AST caused by propolis compared to the control. Interestingly, CCl_4_ did not cause significant changes in the parameters compared to the control group with concomitant administration of propolis. Furthermore, propolis caused a considerable amelioration of all parameters compared to the CCl_4_ treated group.

**Table 1:** Effect of propolis on CCl4-induced hepato-renal toxicity.

Variables	Interventions				F/p-value

	Control	CCl_4_	Propolis	Propolis+CCl_4_	
Alanine transaminase (U/L)	30.3±3.8	55.6±6.7^*^	29.4±3.8^#^	36.4±4.8^#^	36.81/0.00
Aspartate transaminase (U/L)	73.1±7.7	126.4±7.3^*^	55.6±1.9^*#^	59.9±5.8^#^	110/0.00
Alkaline phosphatase (U/L)	222.2±19.4	374.7±17.6^*^	245.2±16.1^#^	269.5±19.4^#^	83/0.00
Creatinine (mg/dL)	65.2±6.9	127.5±11.3^*^	73.1±10.9^#^	74.7±7.4^#^	56.42/0.00
Blood urea (mg/dL)	48.8±4.8	78.0±8.8^*^	54.6±4.4^#^	55.9±5.1^#^	26.22/0.00
Uric acid (mg/dL)	2.4±0.3	3.9±0.4^*^	2.0±0.3^#^	2.5±0.3^#^	38.32/0.00

* p<0.05 compared to the control group,

# p<0.05 compared to the CCl_4_ group. CCl_4_=Carbon tetrachloride

### The effect of propolis and CCl_4_ on oxidant and antioxidant parameters in kidney

In the CCl_4_ treated group, CCl_4_ caused a significant elevation of kidney MDA, PCO, and AOPP compared to the control group (Table-2). There was no significant difference in MDA, PCO, and AOPP in the kidney tissue between the propolis treated and control groups. Interestingly, when the rats were treated with propolis, CCl_4_ did not cause significant changes in the kidney MDA, PCO, and AOPP compared to the control. However, propolis caused a considerable amelioration of all the oxidative parameters measured in the kidney tissues compared to the CCl_4_ treated group. Regarding antioxidant parameters, propolis did not cause significant changes in CAT, GSH, and ascorbic acid (AA); however, it caused a statistically significant elevation of GPx compared to the control group. CCl_4_ caused a statistically significant lowering of CAT, GSH, GPx, and AA (p<0.05). Interestingly, propolis caused a considerable amelioration of CCl_4_-induced deterioration of antioxidant parameters.

**Table 2:** Effect of the interventions on the oxidative parameters in the kidney of rats.

Variables in the kidney	Interventions				F/p-value

	Control	Propolis	CCl_4_	Propolis+CCl_4_	
Malondialdehyde (nmol/g tissue)	79.39±8.92	88.38±6.89	144.03±12.88*	116.02±13.28*^#^	45/0.00
Protein carbonyl formation (µmol/mg prot)	4.76±0.40	4.40±0.54	7.33±0.53*	5.52±0.68^#^	37/0.00
Advanced oxidation protein products (nmol/mg prot)	0.29±0.05	0.22±0.02*	0.40±0.05*	0.31±0.02^#^	22/0.00
Catalase (mmol H_2_ O_2_/mg prot)	8.06±0.56	7.61±0.84	4.95±0.23*	5.80±0.26*^#^	45/0.000
Glutathione peroxidase (nmol GSH/min/mg prot)	26.25±3.72	31.70±3.8*	18.67±1.24*	26.13±2.11#	20.28/0.00
GSH (mg/g tissue)	7.80±0.19	8.90±0.86	5.23±0.99*	7.80±0.85^#^	23.48/0.00
Ascorbic acid (mg/g tissue)	164.83±8.23	162.55±14.82	109.32±8.42*	143.12±8.83*^#^	35.9/0.00

* p<0.05 compared to the control group,

# p<0.05 compared to the CCl_4_ group. CCl_4_=Carbon tetrachloride, GSH=Reduced glutathione

### The effect of propolis and CCl_4_ on oxidant and antioxidant parameters in liver

In the CCl_4_ treated group, CCl_4_ caused a significant elevation of liver MDA, PCO, and AOPP compared to the control group (Table-3). There was no significant difference between the propolis treated group and the control group in MDA, PCO, and AOPP. Interestingly, when the rats were treated with propolis, CCl_4_ did not cause a significant elevation in the liver MDA, PCO, and AOPP compared to the control. Furthermore, propolis caused a considerable amelioration of all oxidative parameters measured in the liver tissues compared to the CCl_4_ treated group. Regarding antioxidant parameters, propolis did not cause statistically significant changes in GSH, but it significantly increased CAT, GPX, and AA. CCl_4_ caused a statistically significant lowering of CAT, GSH, GPx, and AA (p<0.05). Propolis caused a considerable amelioration of CCl_4_-induced deterioration of antioxidant parameters in the liver tissue.

**Table 3:** Effect of the interventions on the oxidative parameters in the liver of rats.

Variables in the liver	Interventions				F/p-value

	Control	Propolis	CCl_4_	Propolis + CCl_4_	
Malondialdehyde (nmol/g tissue)	64.03 ± 6.65	29.23 ± 1.74*	93.04 ± 8.86*	44.50 ± 6.83	108/0.00
Protein carbonyl formation (µmol/mg prot)	4.26 ± 0.55	3.44 ± 0.14*	7.14 ± 0.84*	2.75 ± 0.11*^#^	84.4/0.00
Advanced oxidation protein products (nmol/mg prot)	0.05 ± 0.00	0.04 ± 0.01*	0.08 ± 0.02*	0.05 ± 0.01^#^	12/0.00
Catalase (mmol H_2_ O_2_/mg prot)	8.17 ± 1.16	12.14 ± 1.50*	3.72 ± 0.61*	10.50 ± 1.16*^#^	51.7/0.00
Glutathione peroxidase (nmol GSH/min/mg prot)	2.48 ± 0.15	3.13 ± 0.35*	1.90 ± 0.10*	2.78 ± 0.17^#^	33.3/0.00
GSH (mg/g tissue)	7.10 ± 1.59	8.62 ± 1.30	3.90 ± 0.02*	6.56 ± 1.04^#^	17.5/0.00
Ascorbic acid (mg/g tissue)	165.22 ± 7.41	189.28 ± 9.98*	113.38 ± 11.99*	150.49 ± 6.93*^#^	71.1/0.00

* p < 0.05 compared to the control group,

# p < 0.05 compared to the CCl_4_ group. CCl_4_ = Carbon tetrachloride,

GSH = Reduced glutathione

### Comparison between liver and kidney oxidant and antioxidant parameters

In the control rats, the kidney content of MDA, AOPP, and GPX was significantly higher than liver (p<0.05) (Table-4). Propolis caused a significant elevation of GPx in the kidney tissue and CAT, AA, and GPx in the liver tissue (Tables-2 and 3). CCl_4_ caused a statistically significant lowering of CAT, GSH, GPX, and AA in the kidney and liver tissues. However, the concomitant administration of the propolis with the CCl_4_ significantly ameliorated the level of CAT, GSH, GPX, and AA in both kidney and liver tissues compared to the CCl_4_ treated groups (p<0.05).

**Table 4:** Comparison between liver and kidney content of oxidative stress parameters in the control.

Variables	Organs		p-value

	Kidney	Liver	
*Malondialdehyde* (nmol/g tissue)	79.39±8.92	64.03±6.65*	0.007
Protein carbonyl formation (µmol/mg prot)	4.76±0.40	4.26±0.55	0.101
Advanced oxidation protein products (nmol/mg prot)	0.29±0.05	0.05±0.00*	0.0001
Catalase (µmol H_2_ O_2_/mg prot)	8.06±0.56	8.17±1.16	0.853
Glutathione peroxidase (nmol GSH/min/mg prot)	26.25±3.72	2.48±0.15*	0.001
GSH (µg/g tissue)	7.80±0.19	7.10±1.59	0.283
Ascorbic acid (µg/g tissue)	164.83±8.23	165.22±7.41	0.769

* p<0.05 compared to the kidney content of oxidative stress parameters in the control. GSH=Reduced glutathione

## Discussion

**Figure-1:**
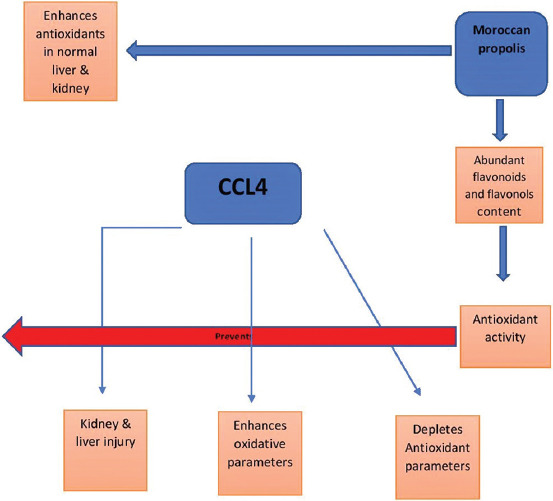
The main effect of propolis in carbon tetrachloride toxicity [Figure prepared by Ahmed Al-Waili].

The data presented demonstrated for the 1^st^ time that Moroccan propolis exhibits a considerable protective effect against CCl_4_-induced liver and kidney damage and significantly improves the deterioration in the antioxidant defense system in the kidney and liver caused by CCl_4_ administration (Figure-1). The chemical analysis showed that the ethanolic extract of propolis contains a high content of flavones, flavanols, and phenolic compounds and has significant antioxidant activity.

The antioxidant content is different in different propolis samples. We have found that Moroccan propolis collected from another area of Morocco (Outat El Haj) contains a lesser quantity of phenols (871.4±17.1 mg GAE100/g) and a higher amount of flavone and flavonol (378.3±11 mg QE/g) [37]. The total phenolic content in Egyptian propolis extracts was 137.52±0.003 mg GAE/g, in Chinese propolis was 123.08±0.005 mg GAE/g, and in Finnish propolis ranges from 79.8 to 156.3 mg/g [38,39]. In Portugal, the total phenol content was 329.00 mg GAE/g in Bornes and 151.00 mg GAE/g in Fundao [40]. Therefore, the total phenolic content in Moroccan propolis was higher than Egyptian, Finnish, Chinese, and Thailand propolis.

The results showed that CCl_4_ causes significant damage in the liver and kidney, which was evident by the elevation of liver enzymes and kidney function parameters. These changes are most likely related to the deterioration of the antioxidant system observed in the CCl_4_-treated rats. Furthermore, the decrease in the antioxidant parameters encountered after CCl_4_ administration might be due to the overuse of the antioxidants by liver and kidney cells to eliminate free radicals [41]. The pretreatment of rats by propolis enhanced antioxidant activity and might make it more effective in removing free radicals.

CCl_4_ is a toxic substance used to induce liver and kidney damage in rats using the cytochrome P-450 system to produce trichloromethyl free radical (CCl_3_) [42]. The free radical combines with cellular lipids and proteins, leading to cellular damage. Although the mechanism of CCl_4_ renal toxicity is almost the same as that of the liver, CCl_4_ shows a higher affinity to the kidney cortex, which is rich in cytochrome P-450 [43]. The pathology of liver damage includes inflammation, necrosis, and fibrosis [44]. Oxidative stress causes cytokine release, cyclooxygenase-2, and interleukin-6, which plays an essential role in liver damage [45].

CCl_4_ caused a significant increase in ALT, AST, ALP, creatinine, BU, and UA, and the previous studies showed a similar effect [46,47]. It is well-known that a high plasma level of liver enzymes indicates cellular liver damage [48]. Inflammation and cytokines, TNF-α and IL-1β, play a role in CCl_4_-induced hepatic injury [49,50]. The use of anti-TNF-α antibodies attenuates hepatic damage [51]. Furthermore, CCl_4_ increased the infiltration of macrophages and neutrophils during hepatocyte injury [52]. These studies showed the importance of the inflammatory process in liver damage caused by CCl_4_.

CCl_4_ increased oxidative stress and caused cellular death *in vitro* [53]. Elevation of oxidative parameters such as MDA (lipid peroxidation marker) and lowering of SOD and CAT cause kidney and liver injury [54,55]. Furthermore, the impairment of the antioxidative defense system by CCl_4_ causes liver injury by oxidizing the cell’s membrane [56,57]. SOD is an enzyme that catalyzes the superoxide radical to oxygen and H_2_O_2_, and scavenges superoxide anion free radicals, and protects cells. Another study found that CCl_4_ diminished the activity of SOD [55]. Myeloperoxidase, which represents the function of polymorphonuclear neutrophils, induces the generation of free radicals by producing hypochlorite by H_2_O_2_ and chloride ion causing cellular damage [58]. CCl_4_ increases myeloperoxidase [55]. Increased GSH/GSSG ratio maintains immune function and detoxification [59]. The present data demonstrate that Moroccan propolis significantly ameliorates the elevation of the antioxidant parameters and shows the beneficial effect of propolis in the prevention of hepato-renal damage.

The administration of CCl_4_ increased MDA, PCO, and AOPP levels in the liver and kidney tissues, which agrees with the previous studies [5,60]. MDA elevation in the kidney and liver tissues indicated an elevation of lipid peroxidation, which lowered the activity of antioxidant enzymes [61]. However, treatment by propolis significantly decreased the MDA concentration in the kidney and liver tissues. Furthermore, propolis restoring the antioxidant parameters in the renal and hepatic tissues might prevent CCl_4_ tissue from being damaged by its antioxidative capacity.

CCl_4_ significantly lowered antioxidant parameters, including CAT, GPx, AA, and GSH in the kidney and liver tissues. These findings are in agreement with the other studies [42,62]. The use of propolis alleviated the deterioration of antioxidant parameters caused by CCl_4_. Flavonoids might cause direct capture of reactive oxygen species, chelate transition metals such as iron and copper, or inhibit the activity of enzymes responsible for the production of reactive oxygen species [63].

The mechanism of action of propolis is most likely due to antioxidant capacity and anti-inflammatory activity. It decreased oxidative stress markers as well as enhanced antioxidant enzymatic and nonenzymatic parameters. These effects might be due to a large amount of flavonoid and phenol compounds present in propolis. The antioxidant enzymes, CAT and PGx, scavenge the free radicals, and propolis enhances the activity. Chrysin, a flavonoid that presents in propolis, inhibits TNF-α-converting enzyme activity and the inflammation in CCl_4_-induced liver toxicity [64]. Pinocembrin, a flavanol found in propolis, has anti-inflammatory and antioxidant effects, enhances the antioxidant defense system, modulates NF-kB and TGF-b1/Smad signaling pathway, and activates Nrf2-mediated HO-1 expression [65].

## Conclusion

Moroccan propolis is a promising source of bioactive compounds such as phenolic acid and flavonoids. It has a high antioxidant activity and a significant protective effect against liver and kidney damage caused by the CCl_4_. Although the exact mechanism of its impact is not fully understood, the mechanism could involve reinforcing the enzymatic and nonenzymatic antioxidant defense system.

## Authors’ Contributions

RE, ZK, MM, and BL: Designed the experimental protocols and participated in the experimental work and writing of the manuscript. NA: Analyzed the data and results and wrote the manuscript. AA: Did the statistical analysis. All authors read and approved the final manuscript.
